# Supplementary material for: Cesarean delivery surgical techniques in Africa: A survey study from Ethiopia
Source: PLoS One. 2023 Oct 9;18(10):e0292382. doi: 10.1371/journal.pone.0292382 (PMC10561831; doi:10.1371/journal.pone.0292382)
Supplement: Attachment — Submitted filename: FINAL PLOS ONE AUTHOR RESPONE.odt [file pone.0292382.s003.odt]

Dear Reviewers,

I would like to thank you very much on behalf of all authors for your extensive review along with the valuable comments, majority of which are well taken as they are and included in the revised version. Please find below listed our point-by-point response to all your comments. 

Reviewer #2: 
Thank you for the revised version of the manuscript. You have addressed the concerns and the manuscript has improved.
Here are just some small suggestions:
Abstract:
L 46: I am not sure of this English is correct. I would say: “Blunt and sharp, cc and lateral, closure and non-closure… “
Response: Thank you very much for making this valuable comment. It has been corrected accordingly. 
Introduction
L. 71 I don’t like the term developed world, it is a bit condescending. I would just say High Income Countries.
Response: Thank you. It has been corrected accordingly. 
L 74 I don’t know what you mean with Among the SSA and Ethiopia representing something. Maybe just remove it.
Response: You are quite right. We mean that Ethiopia as the second largest population in the Sub-Saharan region with high fertility rate, it represents the region with this large burden of obstetric patients and procedures, including CS. We have modified the English a little bit but leaving it as it may be okay. 

Discussion
L 172, recruited, found
Response: Thank you for noting this silly mistake. I have separated the statement with a colon.

In the second paragraph you say there is no difference and in the conclusions you iterate that these options are evidence based. This renders your research a bit pointless. And is the weakest point of the manuscript in my opinion. Why are you researching something that doesn’t matter? Also in the introduction this is not very clear, you just say it is unknown, not why is should be known. Maybe you can argue that practice variation is undesirable in general because it can be confusing when different doctors/hospitals have to work together, even though the technique itself is evidence based. Or for training-purposes. Or at least you can say that the practice variation that you found is consistent with the fact that there is no evidence which technique is better. If all doctors would experience one technique is better you would end practice variation.
Response: Thank you very much for this very constructive comment. I have taken your argument with slight modification and replaced the previous argument. You are right, “Why need our study then?”. I have also modified the statement at the introduction section as you suggested. 

Reviewer #3: 

I read with great interest the Manuscript titled “Cesarean delivery surgical techniques in Africa: a survey study from Ethiopia
” (PONE-D-23-15569R1), which falls within the aim of this Journal.
Response: Thank you so much for appreciating our work.

Authors should consider the following recommendations:
-Manuscript should be further revised by a native English speaker.
Response: Thank you. We have do so accordingly.

-Inclusion/exclusion criteria should be better clarified.
Response: Thank you so much for your comment. I have modified it accordingly. 

-What are the actual clinical implications of this study? it is important to report the results obtained by the authors in the context of clinical practice and to adequately highlight what contribution this study adds to the literature already existing on the topic and to future study perspectives.
Response: Thank you very much for your valuable comment. Apologies, a complete one paragraph was missed from the end in the discussion section. I have included your comment in the revised version. Please check the last paragraph in the discussion section.
Reviewer #4: 
Please clearly explain how contribution the results of your study can provide in the literature
Response: Thank you very much for raising this important comment. As it was also raised by the other reviewer. I have addressed it well in the revised version. Please check the last paragraph in the discussion section.

LİNE 83-86 İN Mat&met the information about Ethiopia should be placed in introduction
Response: Thank you so much. I have moved the statement regarding maternal mortality in Ethiopia to introduction but left the information about CS rate and the Ethiopian society of obstetricians and gynecologists in the methods section. One, there is enough information regarding CS in the country in the introduction and the information regarding ESOG we thought it fits well  in the methods, as it is the source of our data.

Please the advantages of techniques explain clearly in introduction
Response: As our study focused on describing the variation in the techniques without analysis of procedure outcomes (advantages and disadvantages) we didn’t discuss about it in the introduction. However, we have mentioned about it based on available evidence in the discussion, where it fits more, as we need to have a say regarding the implication of the findings. 
Please clearly explain which procedures related CS were evaluated during the survey questionnaire such as antiseptics for the preparation of skin, the prophylactic antibiotics choices
Response: Thank you so much for flagging this important comment. I have added as a statement in bracket in the methods and materials section. Please check in the third paragraph. 
There is no statistical analysis information in mat&met
Response: Thank you. The statistical analysis, which a simple descriptive analysis on SPSS version 22 is well described in the methods section as well as in the abstract section. 

Please explain the advantages and disadvantages of each techniques in discussion when you presented the results of literatures related to each techniques
Response: Thank you so much once again for another important comment. We have included the available evidence on this topic in the discussion technique. Please check paragraph 2 and 4 in the discussion section. We couldn’t provide further information as these are the only available studies that compared outcomes of the techniques.
