# Supplementary material for: Cesarean delivery surgical techniques in Africa: A survey study from Ethiopia
Source: PLoS One. 2023 Oct 9;18(10):e0292382. doi: 10.1371/journal.pone.0292382 (PMC10561831; doi:10.1371/journal.pone.0292382)
Supplement: Attachment — Submitted filename: THIRD CD AUTHOR response.odt [file pone.0292382.s004.odt]

Dear Reviewers,

Thank you very much again for your valuable comments. Please find below listed the authors response your comments.

Reviewer #3
 
-Inclusion/exclusion criteria should be better clarified.
Response: Thank you very much. We have modified the inclusion and exclusion criteria accordingly.
-Manuscript should be further revised by a native English speaker
Response: Thank you very much. The manuscript has been edited extensively by a native English speaker.
Reviewer #4
There is no additional comment. the authors have adequately addressed mycomments. it can be accepted with revison version
Response: Thank you very much!
